# New Triterpenoid from Novel Triterpenoid 15-*O*-Glycosylation on Ganoderic Acid A by Intestinal Bacteria of Zebrafish

**DOI:** 10.3390/molecules23092345

**Published:** 2018-09-13

**Authors:** Te-Sheng Chang, Chien-Min Chiang, Tzi-Yuan Wang, Chun-Hsien Lee, Yu-Wen Lee, Jiumn-Yih Wu

**Affiliations:** 1 Department of Biological Sciences and Technology, National University of Tainan, No. 33, Sec. 2, Shu-Lin St., Tainan 70005, Taiwan; mozyme2001@gmail.com (T.-S.C.); derekww2719@gmail.com (C.-H.L.); s10458017@gm2.nutn.edu.tw (Y.-W.L.); 2 Department of Biotechnology, Chia Nan University of Pharmacy and Science, No. 60, Sec. 1, Erh-Jen Rd., Jen-Te District, Tainan 71710, Taiwan; cmchiang@mail.cnu.edu.tw; 3 Biodiversity Research Center, Academia Sinica, Taipei 115, Taiwan; tziyuan@gmail.com; 4 Department of Food Science, National Quemoy University, No. 1, University Rd., Jin-Ning Township, Kinmen County 892, Taiwan

**Keywords:** biotransformation, *Ganoderma lucidum*, ganoderic acid A, *Bacillus*

## Abstract

Functional bacteria that could biotransform triterpenoids may exist in the diverse microflora of fish intestines. Ganoderic acid A (GAA) is a major triterpenoid from the medicinal fungus *Ganoderma lucidum*. In studying the microbial biotransformation of GAA, dozens of intestinal bacteria were isolated from the excreta of zebrafish. The bacteria’s ability to catalyze GAA were determined using ultra-performance liquid chromatography analysis. One positive strain, GA A07, was selected for functional studies. GA A07 was confirmed as *Bacillus* sp., based on the DNA sequences of the 16S rRNA gene. The biotransformed metabolite was purified with the preparative high-performance liquid chromatography method and identified as GAA-15-*O*-β-glucoside, based on the mass and nuclear magnetic resonance spectral data. The present study is the first to report the glycosylation of *Ganoderma* triterpenoids. Moreover, 15-*O*-glycosylation is a new microbial biotransformation of triterpenoids, and the biotransformed metabolite, GAA-15-*O*-β-glucoside, is a new compound.

## 1. Introduction

*Ganoderma lucidum* is known to the Chinese as “Lingzhi”, which has been used as a diet therapy in traditional Chinese medicine for more than 2000 years. *G. lucidum* is used in the prevention or treatment of many diseases, especially for immunomodulatory and antitumor activities [1]. *G. lucidum* contains many bioactive components, where polysaccharides and triterpenoids are the main bioactive components. Until now, more than 300 different triterpenoids have been identified from *Ganoderma* spp. [2]. All *Ganoderma* triterpenoids are the tetracyclic lanostane type. Among the *Ganoderma* triterpenoids, ganoderic acid A (GAA) was the first identified [3]. GAA is found only in *Ganoderma*; therefore, GAA can be used as a marker component for evaluating *Ganoderma* quality. GAA has been identified with some bioactivities, including antitumor [4,5,6], anti-inflammatory [7], antinociceptive [8], and antioxidative [9] activities.

Finding a new compound is the first step in the development of new drugs. In addition to synthetic and natural sources, microbial biotransformation is an alternative route to obtaining new compounds. Xenobiotics could be catalyzed by microorganisms to form new compounds. Moreover, modifications of the functional groups of the precursors through microbial biotransformations sometimes improve the bioactivities of the precursor compounds. Due to the many bioactivities of triterpenoids, some researchers have focused on the study of triterpenoid biotransformations to search for novel bioactive triterpenoids [10,11,12,13]. Among various biotransformations, glycosylation (the attachment of a bulky sugar group to triterpenoids) could improve physical and chemical stability and aqueous solubility and reduce the cytotoxicity of natural triterpenoids. Increasing aqueous solubility and decreasing cytotoxicity could expand the applications of triterpenoids in advance. Microflora of fish intestines appear to vary with the complexity of the fish digestive system [14]. Dominant members of the γ subclass of Proteobacteria (mainly *Aeromonas* and Enterobacteriaceae), the beta subclass of Proteobacteria, and Gram-positive bacteria with high and low DNA G + C content, are found in the gastrointestinal tracts of fishes [15,16,17]. Some bacteria have been found to be beneficial microbes in aquaculture [18,19]. Taken the above together, we are interested in investigating the biotransformation of *Ganoderma* triterpenoids and focused on the glycosylation biotransformation of intestinal bacteria. In the present study, intestinal bacteria were isolated, and the ability of the bacteria to catalyze GAA was determined. One positive strain was selected and then identified with genetic analysis. The biotransformed metabolite was purified with preparative high-performance liquid chromatography (HPLC) and identified using spectra methods.

## 2. Results

### 2.1. Screening and Identification of Intestinal Bacteria with Biotransformation Activity

**Figure 1:**
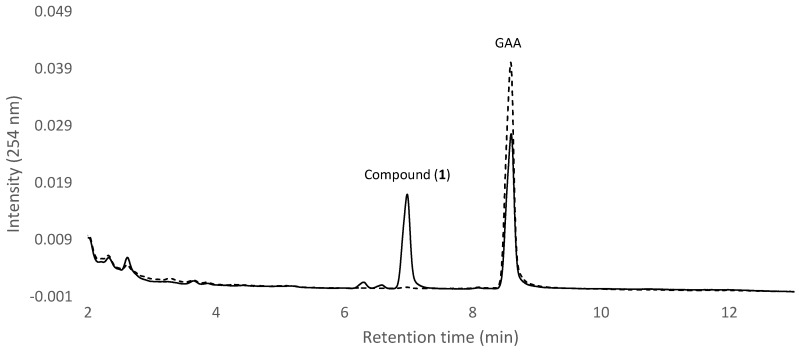
Biotransformation of ganoderic acid A (GAA) using the intestinal isolated bacteria GA A07 strain. The strain was cultivated in Luria-Bertani (LB) media containing 100 mg/L of GAA. The initial (dashed curve) and 24 h (solid curve) cultivations of the fermentation broth were analyzed with ultra-performance liquid chromatography (UPLC). The UPLC operation conditions are described in the Materials and Methods.

To study the biotransformation of *Ganoderma* triterpenoid GAA, dozens of intestinal bacteria were isolated with the plating method and then cultivated in broth with GAA. The fermentation broth was analyzed using ultra-performance liquid chromatography (UPLC) to determine the ability of the strain to digest GAA. A total of 83 strains were screened, and one strain (GA A07) was selected for functional studies. Figure 1 shows the UPLC analysis of initial (dashed curves) and 24 h (solid curve) fermentation broths of the strain in the presence of 100 mg/L GAA. In the figure, GAA with a retention time (RT) of 8.6 min decreased, while one new peak, compound (**1**), with a RT of 7.0 min, appeared after 24 h fermentation. Compound (**1**) did not appear in the 24 h fermentation broth in the absence of GAA (Figure S1). In addition to GAA, antcin K, which is a major ergostane triterpenoid from the fruiting bodies of *Antrodia cinnamomea*, was used as a precursor in this biotransformation study. To evaluate whether the GA A07 strain could also biotransform antcin K, the strain was cultured in the presence of 100 mg/L of antcin K. After a 24 h cultivation, the fermentation broth was analyzed with UPLC. The results showed that no biotransformed metabolite of antcin K by the strain was observed (Figure S2). From the results, it was concluded that GAA was biotransformed to compound (**1**) by the strain, while antcin K was not biotransformed by the strain.

**Figure 2:**
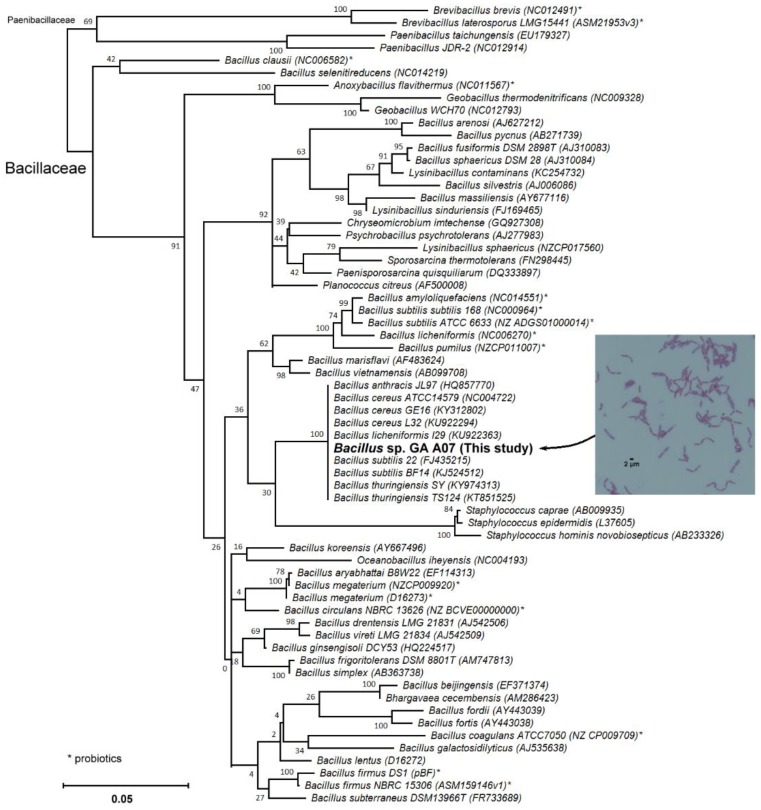
Molecular phylogenetic analysis of the strain GA A07 with the maximum likelihood (ML) method based on the general time reversible (GTR) model [21]. The tree with the highest log likelihood (−8129.86) is shown. The bootstrapping percentage of trees in which the associated taxa clustered together is shown next to the branches [22]. The initial tree(s) for the heuristic search were obtained automatically by applying neighbor-join and BioNJ algorithms to a matrix of pairwise distances, estimated using the maximum composite likelihood (MCL) approach, and then selecting the topology with the superior log likelihood value. A discrete γ distribution was used to model evolutionary rate differences among the sites (five categories (+G, parameter = 0.2049)). The rate variation model allowed for some sites to be evolutionarily invariable ([+I], 56.22% sites). The tree is drawn to scale, with branch lengths measured in the number of substitutions per site. The analysis involved 64 nucleotide sequences. All positions with less than 95% site coverage were eliminated. That is, fewer than 5% alignment gaps, missing data, and ambiguous bases were allowed at any position. There were a total of 1140 positions in the final dataset. Evolutionary analyses were conducted in Molecular Evolutionary Genetics Analysis (MEGA) version X [23]. Gram staining revealed the GA A07 strain is short-rod-shaped, Gram-positive bacteria (see the inserted photo).

To identify the strain, the partial 16S rRNA gene was amplified and sequenced. The partial sequences of the 16S rRNA gene were then blasted against National Center for Biotechnology Information (NCBI) non-redundant nucleotides. From the blasted results, the phylogenetic tree indicated that the GA A07 strain was classified as *Bacillus* sp. (Figure 2). In addition, the GA A07 strain was close to *Bacillus cereus* in the results of the phylogenetic analysis. *Bacillus cereus* is ubiquitous in nature, and most isolates appear to be harmless. However, a few isolates have been identified as the causative agents of anthrax-like severe pneumonia in humans, and these isolates were found to harbor the *Bacillus anthracis* virulence plasmid pXO1 [20]. To determine whether the GA A07 strain possesses the pXO1 plasmid, specific primer sets were used to amplify the conserved sequences of the pXO1 plasmid and the virulent gene, *pag*, on the plasmid. The polymerase chain reaction (PCR) products using primer sets were 512 bp and 596 bp sequences on the pXO1 plasmid, respectively. The results showed that the GA A07 strain did not contain the pXO1 plasmid (Figure S3).

### 2.2. Isolation and Identification of Biotransformation Metabolite

**Figure 3:**
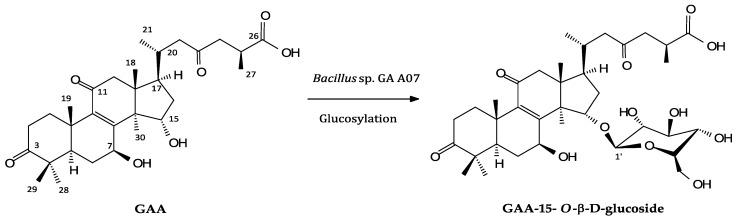
Biotransformation process of GAA using the *Bacillus* sp. GA A07 strain.

To resolve the chemical structure of compound (**1**), the biotransformation was scaled up, and compound (**1**) was purified with preparative high-performance liquid chromatography (HPLC). From a 400 mL fermentation broth containing 40 mg GAA, 24.5 mg of compound (**1**) was isolated. The chemical structures of this metabolite were identified using mass and nuclear magnetic resonance (NMR) spectrum analysis. The mass spectral data showed an [M − H]^−^ ion peak at *m*/*z*: 677.67 in the electrospray ionization mass (ESI-MS) spectrum corresponding to the molecular formula C_36_H_53_O_12_. Then ^1^H and ^13^C-NMR were obtained, and the ^1^H- and ^13^C-NMR signal assignments were conducted, and assisted with spectra of distortionless enhancement, using polarization transfer (DEPT), heteronuclear single quantum coherence (HSQC), heteronuclear multiple bond connectivity (HMBC), ^1^H-^1^H correlation spectroscopy (COSY), and nuclear Overhauser effect spectroscopy (NOESY; shown in Figures S4–S10). Among the 36 signals in the ^13^C-NMR spectrum of compound (**1**), 30 signals corresponding to the triterpenoid structure of GAA [24] were completely assigned, while a significant downfield shift of C-15 (82.7 ppm) and a minor shift of C-6, C-7, C-8, C-17, and C-30 were observed. The other six peaks from 62 to 106 ppm (105.3, 75.9, 78.9, 71.5, 78.6, and 62.7 ppm) indicated the presence of a glucose moiety. The proton signals of glucose were assigned by the ^1^H-^1^H COSY spectrum, and the anomeric proton at δ 4.95 (*J* = 7.9 Hz) in the ^1^H-NMR spectrum indicated the β-configuration of glucopyranosyl moiety. The linkage of glucose to the GAA was demonstrated at the 15-*O* position by cross peaks of H-1′ with C-15 (4.95/82.7 ppm) and H-15 with C-1′ (5.41/105.3 ppm), in the HMBC spectrum. Therefore, compound (**1**) was characterized as GAA-15-*O*-β-glucoside. The key HMBC correlations of compound (**1**) are shown in Figure S11, and the spectroscopic data is listed in Table S1. The biotransformation process of GAA by the *Bacillus* sp. GA A07 strain, where D-glucose was tentatively assumed, is shown in Figure 3. 

## 3. Discussion

Before the present study, three bacteria had been proven to possess glycosylation activity toward triterpenoids [25,26,27,28]. All three bacteria favor catalyzing glycosylation at the carboxyl groups of the triterpenoid precursors. Among these bacteria, *B. subtilis* ATCC 6633 has been demonstrated to glycosylate the C-28 carboxyl group of either the pentacyclic triterpenoids oleanolic acid, echinocystic acid, betulinic acid [26] or the C-26 carboxyl group of the tetracyclic ergostane triterpenoid antcin K [25]. In addition, *Bacillus megaterium* CGMCC 1.1741 [27] and *Nocardia coralline* CGMCC 4.1037 [28] were proven to glycosylate the C-28 carboxyl group of the pentacyclic triterpenoids ursolic acid and echinocystic acid, respectively. Interestingly, in the present study, the *Bacillus* sp. GA A07 strain was proven to glycosylate the C-15 hydroxyl group of GAA. This is a new glycosylation site for biotransformation of triterpenoids. In the GAA biotransformation using the GA A07 strain, only one metabolite, which was glycosylated at the C-15 hydroxyl group, was observed (Figure 1 and Figure 3), although the other C-7 hydroxyl or C-26 carboxyl groups on the GAA structure were available for glycosylation. Moreover, the GA A07 strain could not biotransform antcin K (Figure S2), which contains C-3, C-4, C-7 hydroxyl, and C-26 carboxyl groups, but lacks the C-15 hydroxyl group available for glycosylation in its structure. The results revealed that the GA A07 strain has regioselection in the glycosylation of the triterpenoid. Thus, due to a unique and specific catalyzing site, the GA A07 strain discovered in the present study is valuable in the application of biotransformation of triterpenoid glycosylation.

However, there are three microbial glycosyltransferases (GTs), which, until now, have been identified with triterpenoid glycosylation activity. They are BsYjiC from *B. subtilis* 168 [29,30], UGT109A1 from *B. subtilis* CTCG 63501 [31], and BsGT1 from *B. subtilis* KCTC 1022 [32]. These GTs catalyze either C-3, C-6, C-12, or C-20 carbon positions with a hydroxyl group for the glycosylation of triterpenoids. Accordingly, the enzyme that catalyzes the C-15 glycosylation of GAA in the GA A07 strain is a unique GT. Thus, it is worthwhile to clone the putative gene encoding the enzyme catalyzing the C-15 glycosylation of GAA from the genome of the GA A07 strain. The study was undertaken in our laboratory.

Many *Bacillus* strains were found to be beneficial microbes in aquaculture [19]. For example, feeding diets containing mixed *B. firmus*, *B. pumilus,* and/or *B. subtilis*, *B. licheniformis*, showed better survival rates and growth for fishes [33,34]. The presence of *B. subtilis* C-3102 in the diets of hybrid tilapia juvenile caused upregulation of cytokines, such as interleukin-1β, tumor necrosis factor-β, and tumor necrosis factor-α in the intestines of the fish [35]. Although most *Bacillus* strains are safe, some strains of the *B. cereus*, *B. popilliae*, *B. pumilus*, and *B. subtilis* group species may be still problematic due to enterotoxins and/or an emetic toxin, or associated with food poisoning [36]. Nevertheless, Figure S3 revealed that this strain did not contain the virulence plasmid pXO1, and might be harmless.

Glycosylation is a common modification reaction in the biosynthesis of natural compounds. Glycosylation enhances water solubility and lipophilic compounds. GAA has been identified with some bioactivities, including antitumor activities [4,5,6]. Some reports showed that the bioactivities of triterpenoids were improved through glycosylation of the triterpenoids. Wang et al. [26] found that mono-glycosylation of triterpenoids betulinic acid and oleanic acid can significantly enhance their inhibitory effects on tissue factor procoagulant activity. Moreover, Quan et al. [37] found that the number and positon of sugar linkage in ginsenosides showed strong effects on anticancer activity. Therefore, the new GAA triterpenoid glucoside in the present study might have novel biological activities. Although the biological activities of the new compound have not yet been determined, it might have some medicinally, cosmetically, and pharmacologically important properties. Potential health-benefiting properties of the new compound could help develop future therapeutic agents. Moreover, the development of a new triterpenoid glycosylation biotransformation system could expand to find new *Ganoderma* triterpenoids and applications of the newly produced *Ganoderma* triterpenoids in medicine, cosmetics, and pharmacology in the future.

## 4. Materials and Methods

### 4.1. Microorganism and Chemicals

GAA was purchased from Baoji Herbest Bio-Tech (Xi’An, China). All the materials needed for PCR, including primers, deoxyribonucleotide triphosphate, and Taq DNA polymerase, were purchased from MDBio (Taipei, Taiwan). The other reagents and solvents used were of high quality, and purchased from commercially available sources.

### 4.2. Screening and Identification of Intestinal Bacteria with Biotransformation Activity

The intestinal symbiotic bacteria was isolated from the excreta of cultivated AB zebrafish (*Danio rerio*). Zebrafish excreta were collected from the bottom of fish containers. The excreta were resolved in Luria-Bertani (LB) broth and then plated according to the dilution plating method on LB agar [38]. The screening and identifying methods were modified from our previous report by replacing 100 mg/L of antcin K with 100 mg/L of GAA in LB medium with 50 g/L of glucose. All other screening and identification conditions were maintained.

### 4.3. UPLC Analysis

The UPLC system (Acquity UPLC H-Class, Waters, Milford, MA, USA) was equipped with an analytic C18 reversed-phase column (Kinetex^®^ C18, 1.7 µm, 2.1 i.d. × 100 mm, Phenomenex Inc., Torrance, CA, USA). The operation conditions for the UPLC analysis were from our previous study [38].

### 4.4. Candidate Strain Classification via 16S rRNA Gene Analysis

The GA A07 strain was classified using the genetic analysis according to our previous study [38]. Briefly, the genomic DNA of the GA A07 strain was isolated, and the 16S ribosomal RNA gene sequences of the GA A07 strain were amplified from the chromosomal DNA using PCR with the forward (5′-AGA GTT TGA TCC TGG CTC AG-3′) and reverse (5′-GAC GGG CRG TGW GTR CA-3′) primers known to amplify the 16S rRNA gene from a broad range of taxonomically different bacterial isolates. The sequence of the amplified DNA fragment was determined and then blasted against NCBI non-redundant nucleotides. The 16S rRNA phylogeny was then constructed to classify the strain.

### 4.5. Confirming Possession of the pXO1 Plasmid

PCR was used to determine whether the GA A07 strain possessed the pXO1 plasmid. The specific primer sets were used for amplification of the conserved sequences of pXO1 plasmid and the virulent gene, *pag* [20]. The specific primer sets were the forward (5′-CTA GAA CTT ACT GAT ACG GAG TG-3′) and reverse (5′-TTC AGT ACC TTT TAT CTA CCC A-3′) primers for pXO1, and the forward (5′-TCC TAA CAC TAA CGA AGT CG-3′) and reverse (5′-GAG GTA GAA GGA TAT ACG GT-3′) primers for the *pag* gene. The PCR conditions were also adapted from the method in Chandranaik et al. [20].

### 4.6. Scaled-Up Fermentation, Isolation, and Identification of the Biotransformation Products

The GA A07 strain was cultured in a 1000 mL baffled Erlenmeyer flask containing 100 mL of LB medium with 50 g/L of glucose and 100 mg/L of GAA at 180 rpm, 28 °C for 24 h. A total of 4 flasks of GA A07 cultivation (400 mL) were conducted. After cultivation, the fermentation broth was combined and extracted with 100 mL of ethyl acetate twice. The ethyl acetate fractions were combined and condensed under a vacuum. The residue was resuspended in 100 mL of 50% methanol and then applied in a preparative YoungLin HPLC system (YL9100, YL Instrument, Gyeonggi-do, South Korea). The system was operated according to our previous study [38]. Finally, 24.5 mg of compound (**1**) was obtained, and the structure of the compound was confirmed with NMR and mass spectral analysis. The mass analysis was performed on a Finnigan LCQ Duo mass spectrometer (ThermoQuest Corp., San Jose, CA, USA) with electrospray ionization (ESI). ^1^H- and ^13^C-NMR, DEPT, HSQC, HMBC, COSY, and NOESY spectra were recorded on a Bruker AV-700 NMR spectrometer (Bruker Corp., Billerica, MA, USA) at ambient temperature. Standard pulse sequences and parameters were used for the NMR experiments, and all chemical shifts were reported in parts per million (ppm, δ).

## 5. Conclusions

To date, more than 300 triterpenoids have been isolated and identified from *Ganoderma*. However, rare *Ganoderma* triterpenoids exist in glycoside forms. The present study is the first report about glycosylation of *Ganoderma* triterpenoids. Moreover, the metabolite produced, GAA-15-*O*-β-glucoside, is a new compound via biotransformation using the intestinal bacteria of zebrafish. Thus, the present study develops a novel biotransformation system, which might be applied to numerous *Ganoderma* triterpenoids to create many new *Ganoderma* triterpenoid glucosides in the future.

## Supplementary Materials

The following are available online. Table S1. NMR spectroscopic data for compound (**1**) (in pyridine-d5; 700 MHz). Figure S1. UPLC analysis of the 24 h fermentation broth using the GA A07 strain without the addition of GAA. Figure S2. UPLC analysis of the 24 h fermentation broth using the GA A07 strain with the addition of 100 mg/L of antcin K. Figure S3. Gel electrophoresis of the products from PCR with pXO1 specific primer sets. Figure S4. The ^1^H-NMR (700 MHz, pyridine-d5) spectrum of compound (**1**). Figure S5. The ^13^C-NMR (176 MHz, pyridine-d5) spectrum of compound (**1**). Figure S6. The DEPT-90 and DEPT-135 (176 MHz, pyridine-d5) spectra of compound (**1**). Figure S7. The HSQC (700 MHz, pyridine-d5) spectrum of compound (**1**). Figure S8. The HMBC (700 MHz, pyridine-d5) spectrum of compound (**1**). Figure S9. The ^1^H-^1^H COSY (700 MHz, pyridine-d5) spectrum of compound (**1**). Figure S10. The NOESY (700 MHz, pyridine-d5) spectrum of compound (**1**). Figure S11. The Key HMBC (blue arrows, from ^1^H to ^13^C) correlations of compound (**1**).
