# Supplementary material for: New Triterpenoid from Novel Triterpenoid 15-O-Glycosylation on Ganoderic Acid A by Intestinal Bacteria of Zebrafish
Source: Molecules. 2018 Sep 13;23(9):2345. doi: 10.3390/molecules23092345 (PMC6225395; doi:10.3390/molecules23092345)
Supplement: Supplementary file 1 [file molecules-23-02345-s001.pdf]

Supplementary Materials: New Triterpenoid from Novel Triterpenoid 15-*O*-Glycosylation on Ganoderic Acid A by Intestinal Bacteria of Zebrafish

Te-Sheng Chang [1], Chien-Min Chiang [2], Tzi-Yuan Wang [3], Chun-Hsien Lee [1], Yu-Wen Lee [1] and Jiumn-Yih Wu [4,*]

Table S1. NMR spectroscopic data for compound (**1**) (in pyridine-d_5; 700MHz).

Compound	GAA [(a)]	GAA-15-*O*-β-glucoside Compound (**1**)			
Position	δC	type	δC ,	δH (*J* in Hz)	HMBC
GAA moiety					
1	35.6	CH_2	36.3	3.15, ddd (13.0, 7.5, 5.3)<br>1.54, dt (13.6, 8.4)	H-2, H-19
2	34.3	CH_2	34.8	2.58, m<br>2.53, m	H-1
3	217.3	C	216.0		H-1, H-2, H-28, H-29
4	47.0	C	46.9		H-6, H-28, H-29
5	49.2	CH	49.2	1.74, dd (13.5, 1.9)	H-1, H-6, H-19, H-29
6	29.2	CH_2	30.7	2.14, m<br>2.00, m	H-5, H-7
7	69.1	CH	67.2	5.23, t (8.8)<br>6.74 (OH)	H-5, H-6
8	159.4	C	162.1		H-6, H-7, H-15, H-30
9	140.3	C	140.8		H-1, H-7, H-12, H-19
10	38.1	C	38.2		H-1, H-2, H-5, H-6, H-19
11	199.9	C	199.4		H-12,
12	51.7	CH_2	52.3	2.90, d (16.4)<br>2.66, d (16.1)	H-7, H-18
13	46.7	C	46.5		H-12, H-17, H-18, H-30
14	54.0	C	54.3		H-12, H-15, H-16, H-18, H-30
15	72.3	CH	82.7	5.41, dd (9.4, 6.4)	H-16, H-30, **Glc-H-1′**
16	35.9	CH_2	35.8	2.58, m<br>2.03, m	H-17
17	48.0	CH	49.3	1.83, q (9.4)	H-16, H-18, H-21, H-22
18	17.3	CH_3	17.8	1.14, s	H-12, H-17
19	19.6	CH_3	18.9	1.45, s	H-1, H-5
20	32.7	CH	33.0	2.19, m	H-16, H-17, H-21, H-22,
21	19.8	CH_3	19.7	0.93, s	H-22
22	49.7	CH_2	49.9	2.54, m<br>2.19, m	H-21
23	209.1	C	209.1		H-20, H-22, H-24, H-25
24	46.6	CH_2	47.1	3.04, dd (17.5, 8.6)<br>2.53, m	H-25, H-27
25	34.7	CH	35.6	3.27, m	H-24, H-27
26	179.4	C	178.4		H-24, H-25, H-27
27	17.0	CH_3	17.7	1.34, d (7.3)	H-24, H-25
28	27.4	CH_3	26.9	1.07, s	H-5
29	20.8	CH_3	21.0	1.08, s	H-5
30	19.4	CH_3	22.0	1.49, s	H-15
Glucose moiety					
1′		CH	105.3	4.95, d (7.9)	**H-15**, H-2′,
2′		CH	75.9	4.09, m	H-3′
3′		CH	78.9	4.22, m	H2′, H-4′
4′		CH	71.5	4.24, m	H-3′, H-5′
5′		CH	78.6	3.93, m	H-6′
6′		CH_2	62.7	4.53, m<br>4.38, dd (11.8, 5.2)	H-4′

(a) Koyama, K.; Imaizumi, T.; Akiba, M.; Kinoshita, K.; Takahashi, K.; Suzuki, A.; Yano, S.; Horie, S.; Watanabe, K.; Naoi, Y. Antinociceptive components of Ganoderma lucidum. *Planta Med.* **1997**, 63, 224–227.. Data were recorded in DMSO-d6.

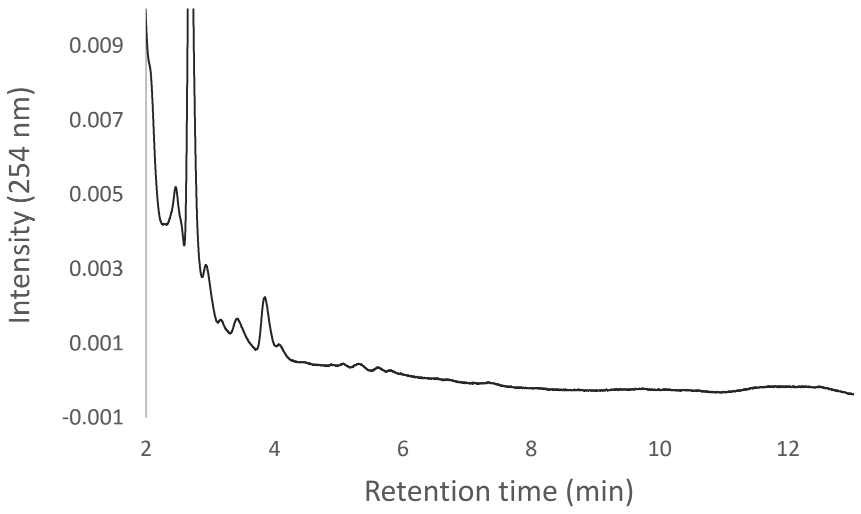

Figure S1. UPLC analysis of the 24 h fermentation broth by GA A07 strain without adding of GAA.

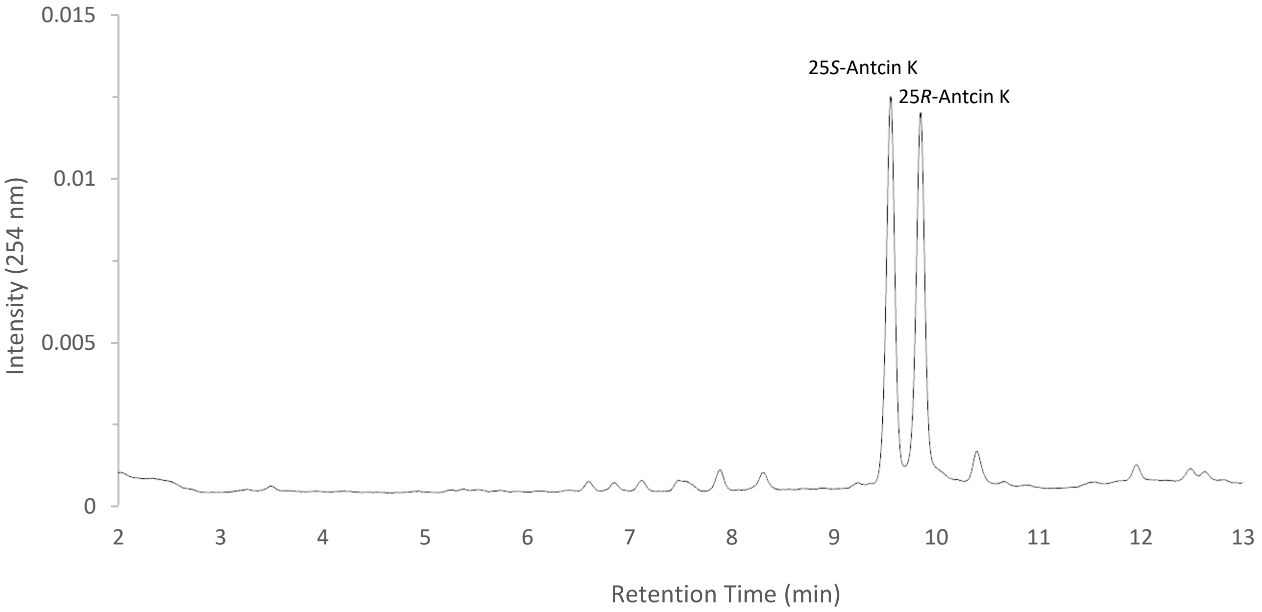

Figure S2. UPLC analysis of the 24 h fermentation broth by GA A07 strain with adding of 100 mg/L of antcin K.

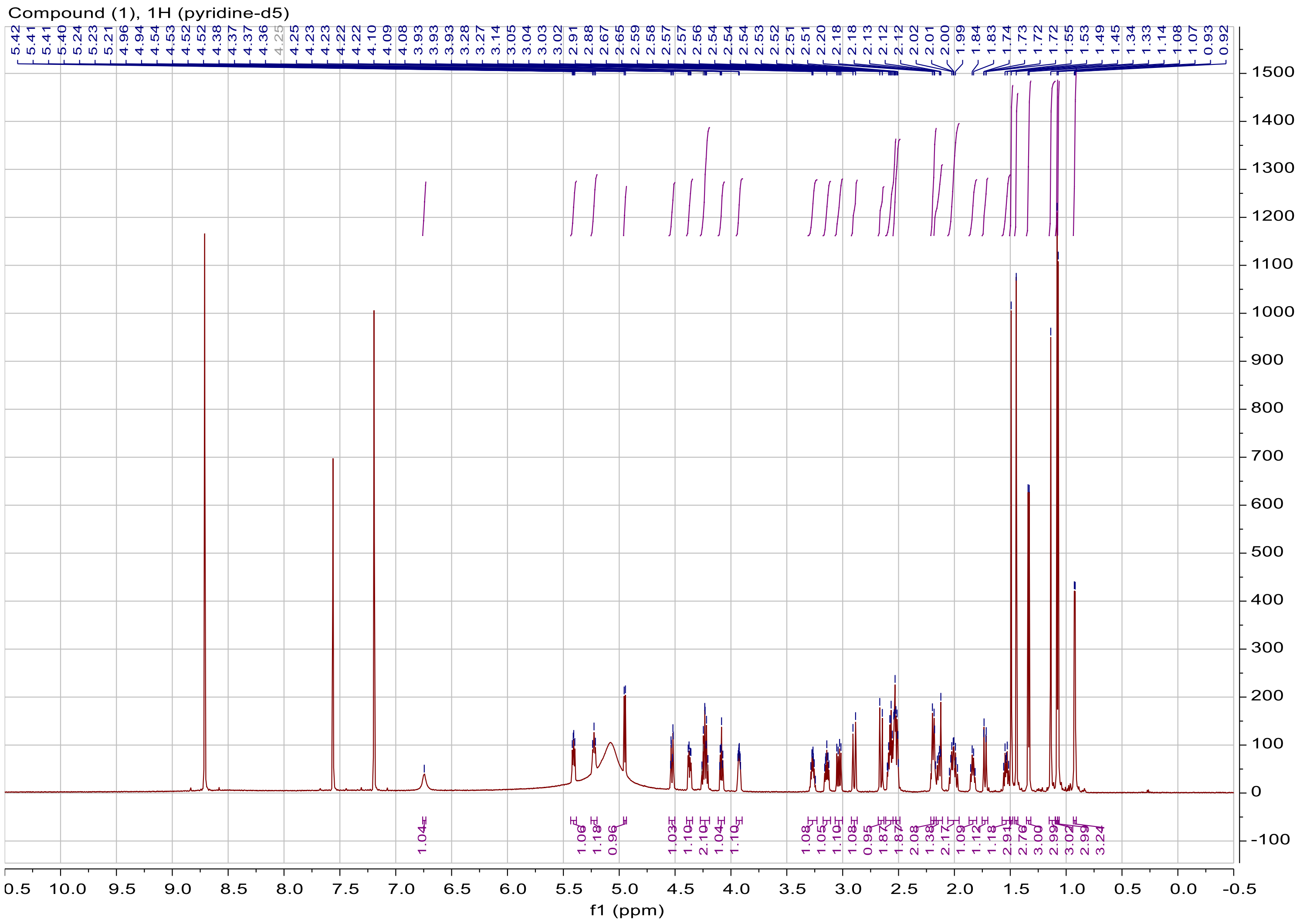

Figure S3. The ^{1}H-NMR (700 MHz, pyridine-*d*$_5$) spectrum of compound (**1**).

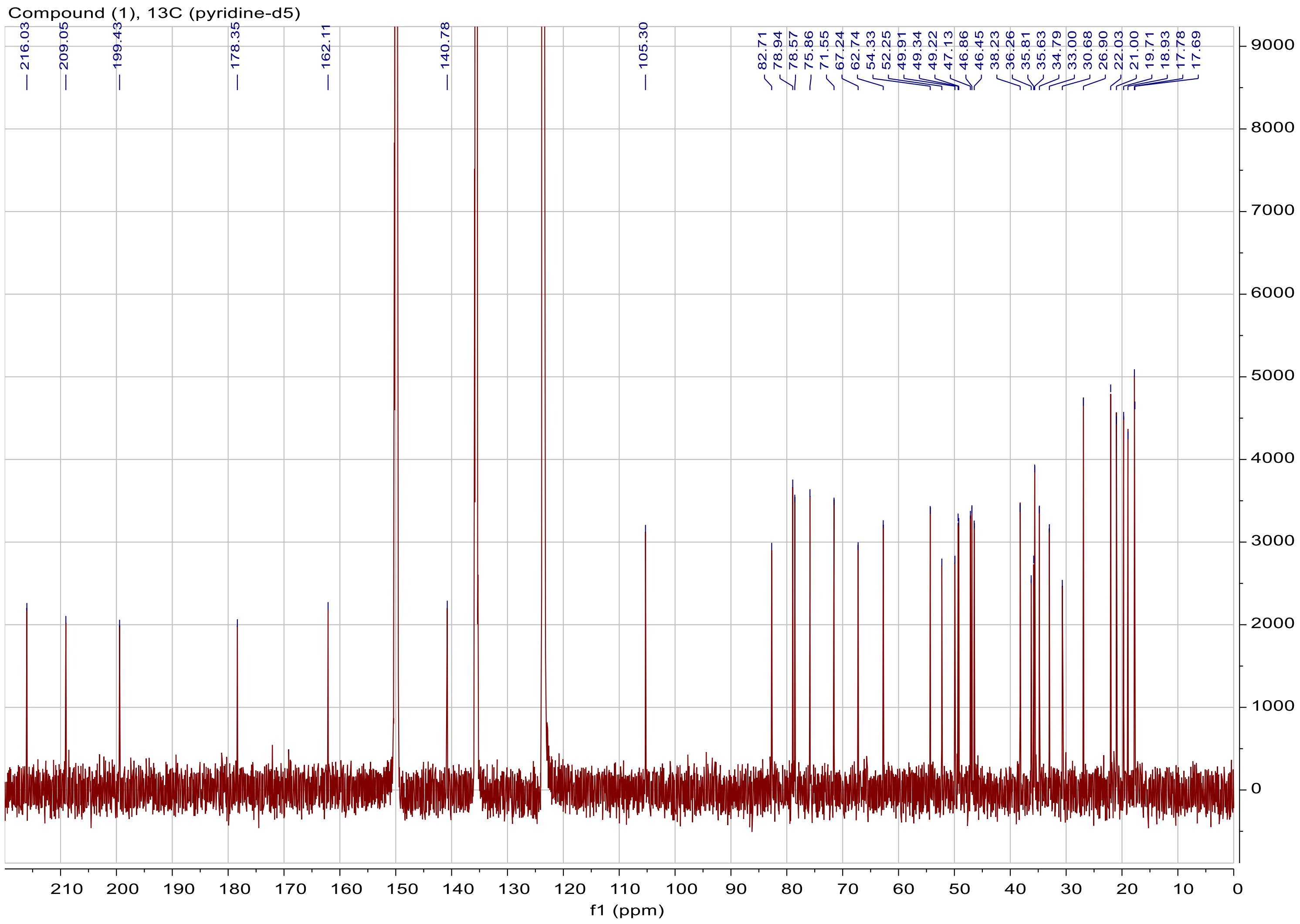

Figure S4. The ^{13}C-NMR (176 MHz, pyridine-d_5) spectrum of compound (**1**).

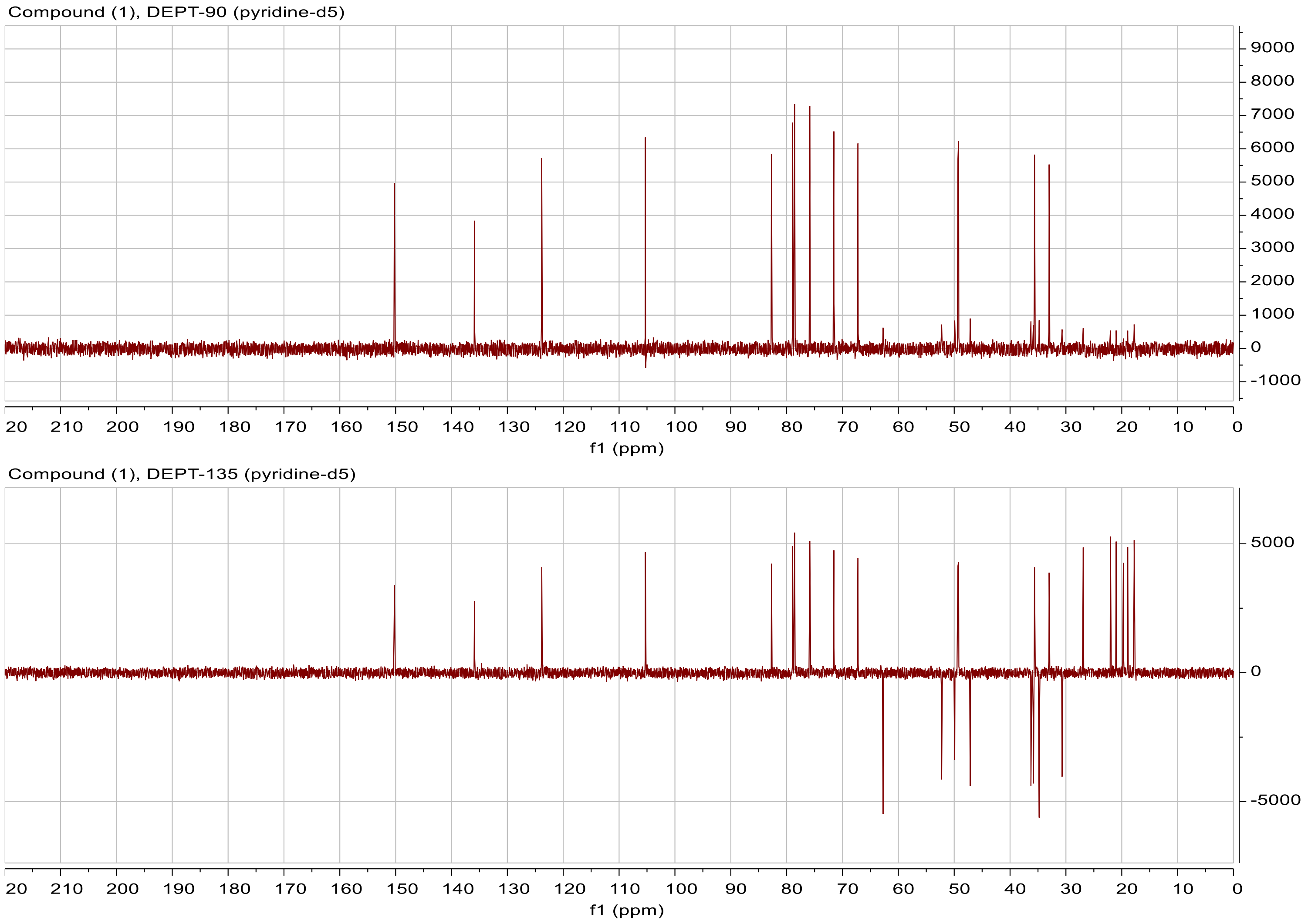

Figure S5. The DEPT-90 and DEPT-135 (176 MHz, pyridine-*d*5) spectra of compound (**1**).

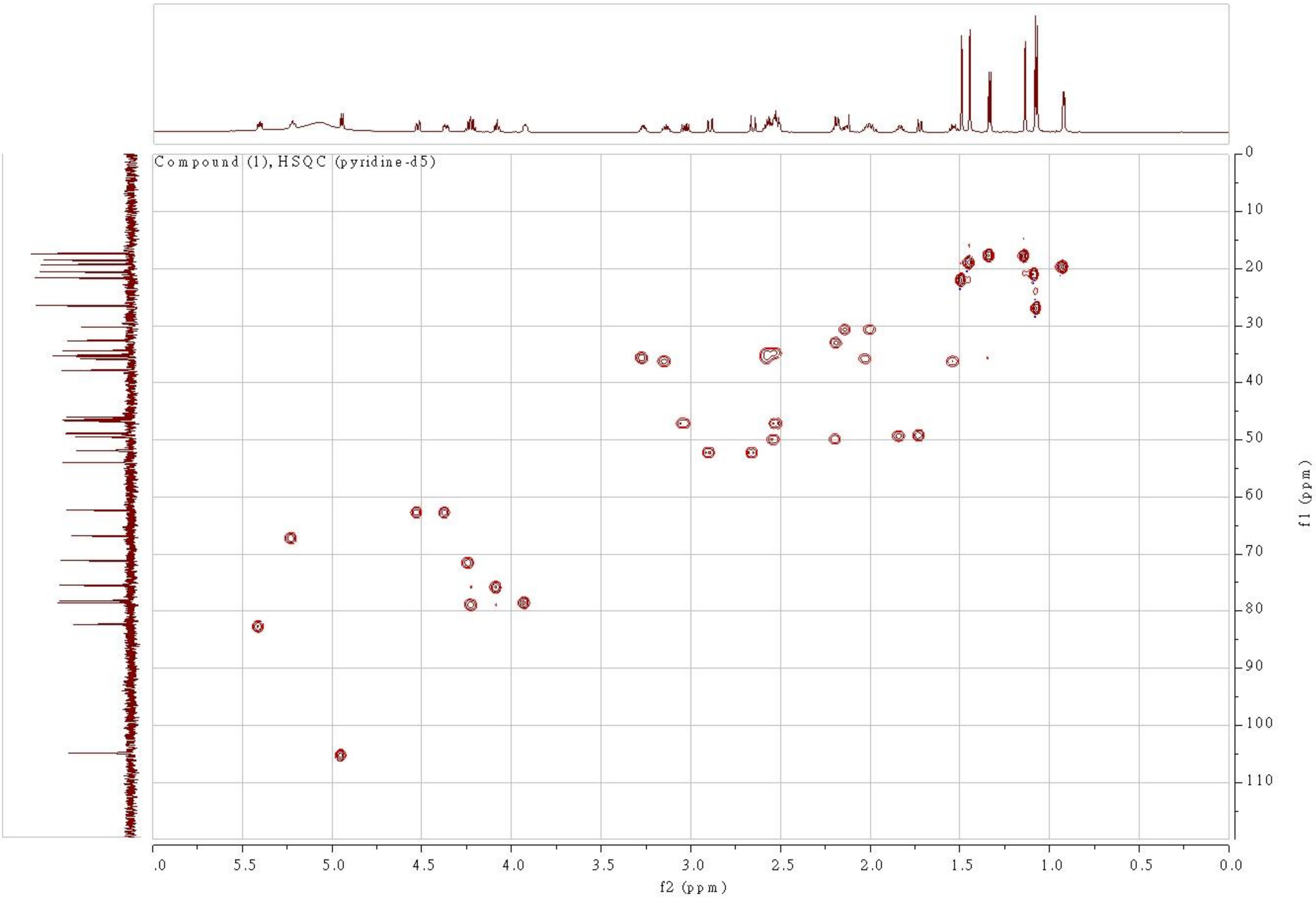

Figure S6. The HSQC (700 MHz, pyridine-d_5) spectrum of compound (**1**).

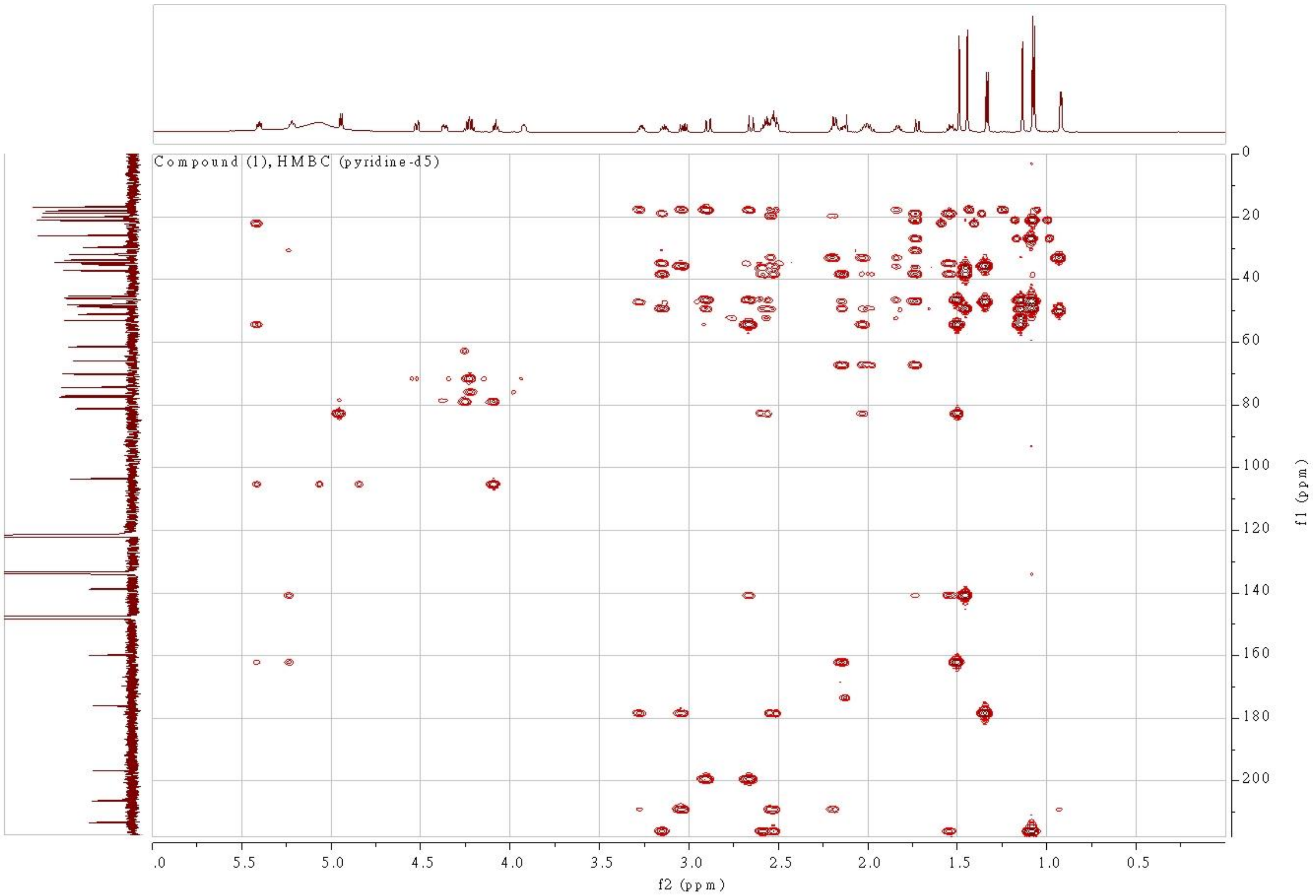

Figure S7. The HMBC (700 MHz, pyridine-*d*5) spectrum of compound (**1**).

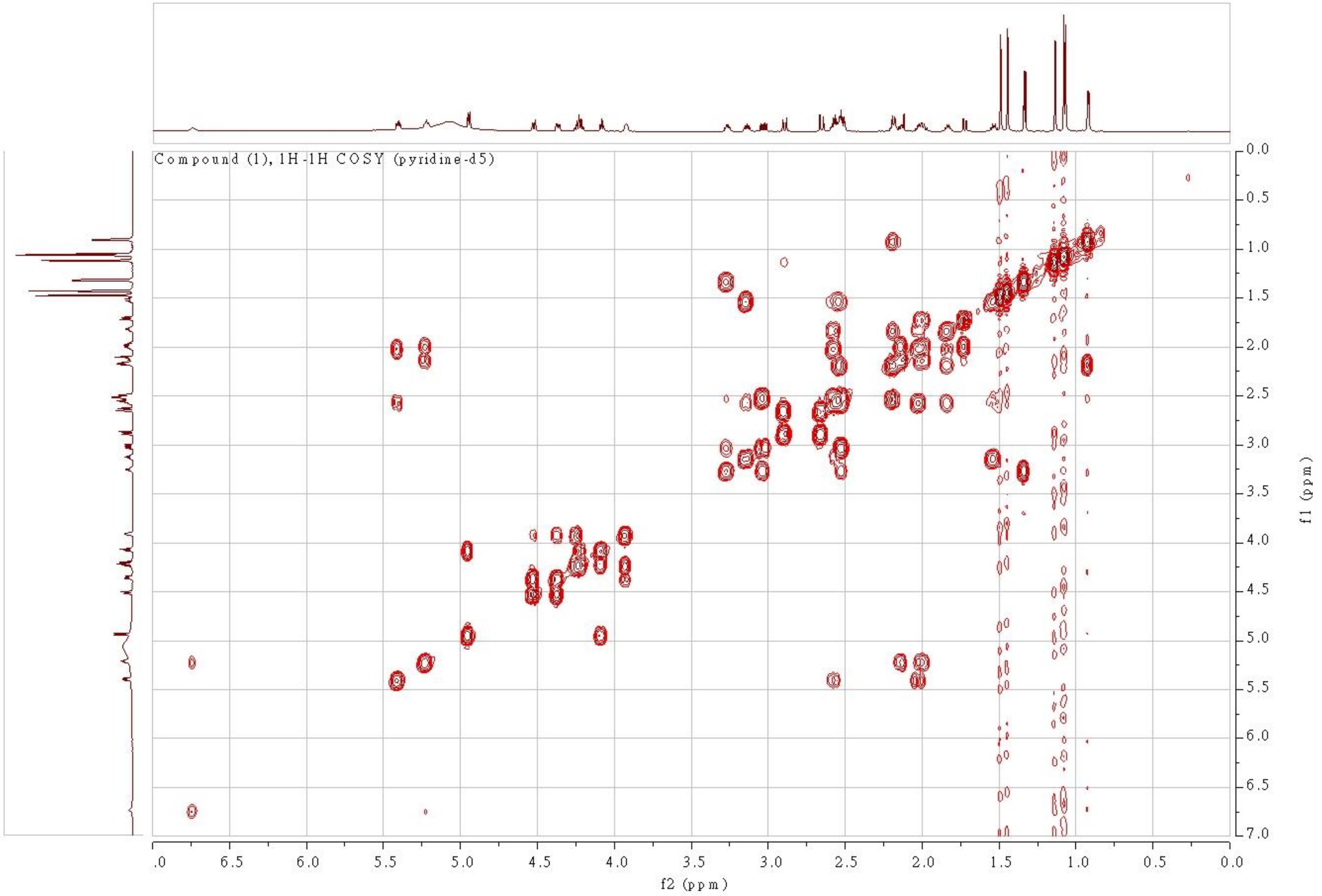

Figure S8. The ^{1}H-^{1}H COSY (700 MHz, pyridine-d_5) spectrum of compound (**1**).

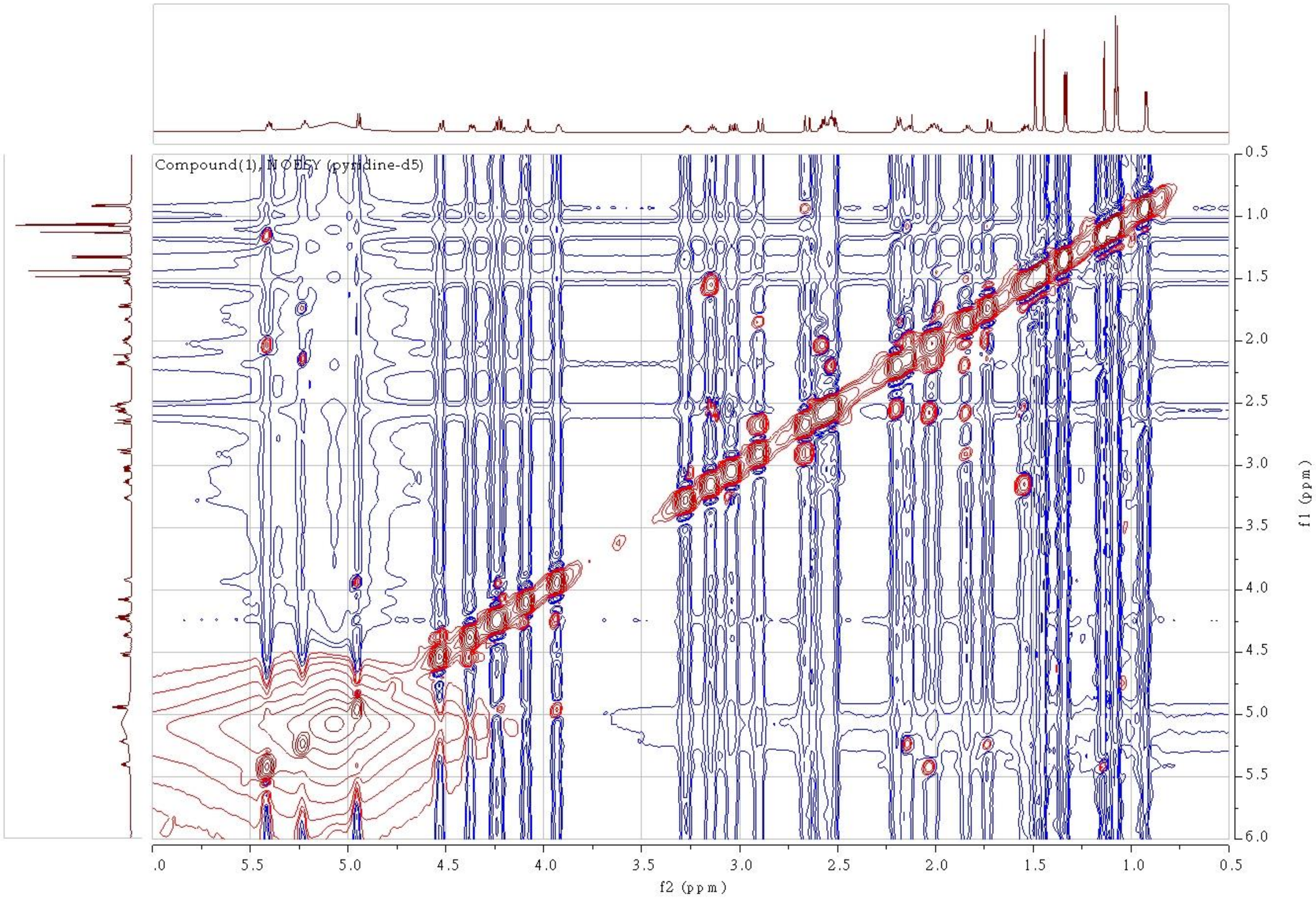

Figure S9. The NOESY (700 MHz, pyridine-d_5) spectrum of compound (**1**).

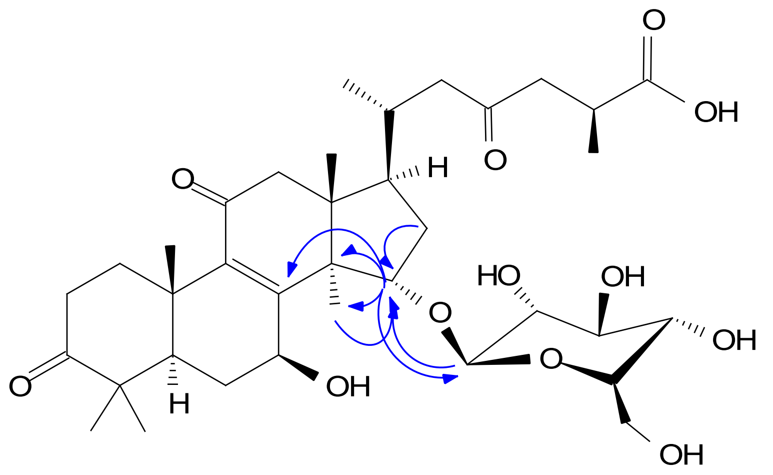

Figure S10. The Key HMBC (blue arrows, ^{1}H to ^{13}C) correlations of compound (**1**).
